# Photoelectrochemical Stability Enhancement of (311)-Oriented Indium Sulfide Thin Films via In-Cystine Complex Formation under Hydrothermal Synthesis

**DOI:** 10.1021/acsaem.5c03482

**Published:** 2026-02-10

**Authors:** Xiuru Yang, Hong Chang, Arthur Graf, Xiaohong Li, Yongde Xia, Asif Ali Tahir, Yanqiu Zhu

**Affiliations:** a Department of Engineering, Faculty of Environment, Science and Economy, 3286 University of Exeter, Exeter EX4 4QF, U.K.; b HarwellXPS, Research Complex at Harwell, Rutherford Appleton Lab, Didcot OX11 0FA, U.K.; c School of Chemistry, Cardiff University Main Building, Park Place, Cardiff CF10 3AT, U.K.; d Renewable Energy Group, Department of Engineering, Faculty of Environment, Science and Economy, University of Exeter, Penryn TR10 9FE, U.K.; e Solar Energy Research Group, Environment and Sustainability Institute, Faculty of Environment, Science and Economy, University of Exeter, Penryn Campus, Penryn TR10 9FE, U.K.

**Keywords:** indium sulfide thin film, photoanode, PEC water splitting, photocurrent stability, In-cystine complex

## Abstract

Indium sulfide is a promising photoactive material for light-induced applications, particularly photoelectrochemical (PEC) water splitting. However, its practical application is limited by photocorrosion, which hinders its long-term efficiency. In this study, we report a hydrothermal synthesis of In-cystine bonded (311)-oriented indium sulfide thin films using a mixed sulfur source of l-cysteine hydrochloride and l-cystine, the latter generated in situ via Fe^3+^-induced oxidation of l-cysteine. Synthesis parameters such as temperature and ramp rate are found to affect the indium-organic complex’s physical and chemical properties such as composition, morphology, thickness, crystal structure, and thereby the PEC performance of the resulting films. The results indicated that thin films synthesized under slow heating conditions (e.g., 160–3 at 160 °C with a ramp rate of 3 °C/min for 6 h; 180–3 at 180 °C with a ramp rate of 3 °C/min for 6 h) exhibited a dominant indium sulfide phase bonded with In-cystine and demonstrated high and stable photocurrent densities of 1.0 and 0.93 mA cm^–2^ at −0.2 V vs Ag/AgCl, respectively. In contrast, the fast-heated thin film 160–10 (prepared at 160 °C with a ramp rate of 10 °C/min for 6 h) primarily contained indium-organic complexes with mixed In-cystine and In-cysteine bonding and exhibited a photocurrent density of 0.35 mA cm^–2^ at −0.2 V vs Ag/AgCl. Stability testing further revealed that after 2 h of continuous illumination at −0.2 V vs Ag/AgCl, the thin film 160–3 retained 0.75 mA cm^–2^, while 180–3 maintained 1.1 mA cm^–2^, demonstrating improved resistance to photocorrosion. This work presents an effective strategy for improving the long-term PEC performance of metal sulfide photoelectrodes by introducing In-cystine bonding at their surface, offering a pathway toward more stable and efficient solar-driven water-splitting devices.

## Graphical abstract

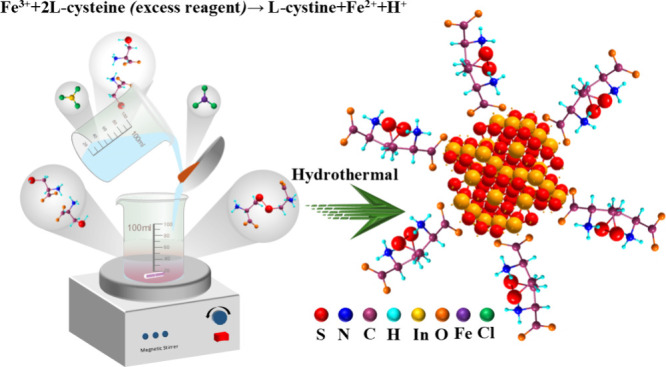

## Introduction

Indium sulfide is an n-type semiconductor with a band gap of 2.0–2.5 eV,^1^ allowing it to respond quickly to light by generating electron–hole pairs upon illumination. This fast photoresponse makes indium sulfide a widely used material in various light-driven applications, including photodetectors,^2,3^ phototransistors,^4,5^ photonic memory devices,^6,7^ ultrafast optical devices in the telecommunications band,^8^ solar cells,^9,10^ photocatalysis,^1,11,12^ and photoelectrochemical (PEC) water splitting.^13–15^ Among all these applications, the role of indium sulfide thin films in PEC water splitting for solar-to-hydrogen conversion contributing to sustainable energy development has been extensively explored. Its suitable band alignment and broad light absorption range enable it to effectively drive the water-splitting reaction. However, like most metal sulfide semiconductors, under light illumination, the accumulation of photogenerated holes at the surface of metal sulfide leads to photocorrosion, where S^2–^ ions in the lattice can be oxidized or leached out, resulting in the loss of PEC activity.^16,17^

To mitigate this issue, a key approach is to prevent the accumulation of holes on the metal sulfides surface.^18^ Two main strategies have been developed: (1) consumption of photogenerated holes to reduce their destructive potential: for example, by using sacrificial reagents Na_2_S and Na_2_SO_3_ ^19–21^ or by construction of Z-scheme heterojunctions like In_2_S_3_/Bi_2_S_3_,^17,22,23^ the valence band of In_2_S_3_ acts as a recombination center for photogenerated electrons from Bi_2_S_3_ and holes from In_2_S_3_; and (2) facilitation of hole transfer away from the In_2_S_3_ surface through heterojunction engineering: for instance, constructing Type II heterojunctions with semiconductors such as g-C_3_N_4_ ^24^ or WO_3_,^25^ which have negative valence band potentials than In_2_S_3_, facilitates the hole transfer from these semiconductors to In_2_S_3_. Other important approaches focus on minimizing direct contact between S^2–^ ions in the metal sulfide lattice and the electrolyte. This can be achieved by controlling the crystallographic orientation of the thin film, for example, by shifting from the (311) facet, which has high sulfur exposure and exhibits higher PEC activity but suffers from poor stability, to the (440) facet, which has lower sulfur exposure, lower activity, but improved stability.^12^ In addition, depositing protective layers such as Al_2_O_3_ or TiO_2_ can isolate the metal sulfide from the electrolyte, thereby mitigating photocorrosion.^20,26^ However, the use of sacrificial reagents is inefficient due to slow diffusion processes, and photogenerated holes preferentially oxidize nearby S^2–^ ions within the lattice. On the other hand, creating heterojunctions or protective layers with secondary semiconductors on the surface of indium sulfide complicates the thin film synthesis. Additionally, controlling the crystal orientation is also particularly challenging. Therefore, it is important to develop a more straightforward approach that can both simplify the thin film synthesis and enhance the PEC stability of metal sulfide thin films.

The amino acid l-cysteine, which has three functional groups, has been widely used as a sulfur source and as a complexing agent for the synthesis of metal sulfides.^27^ In our previous work, we synthesized an In-cysteine-bonded (311)-oriented indium sulfide thin film using l-cysteine hydrochloride. However, these films exhibited poor PEC stability due to the exposure of high-index (311) facets with high sulfur density and the presence of a thiol terminal group in the In-cysteine bonds in these facets.^28^ Both types of sulfur species are unstable in basic environments under illumination, as they are susceptible to photocorrosion.^28^ Notably, Berestova et al. reported that Fe^3+^ can oxidize the l-cysteine to form l-cystine in an acidic medium.^29–34^ The disulfide bond in cystine is more stable than the thiol group, which allows it to act as a protective layer with significant potential to enhance the stability of metal sulfides.^35–37^ In this work, we introduced a controlled amount of FeCl_3_ into the precursor solution to partially oxidize the l-cysteine hydrochloride to l-cystine, creating a mixed sulfur source for the subsequent hydrothermal reaction. We systematically investigated the effects of the temperature ramp rate and synthesis temperature on the phase composition, crystallite size, morphology, and corresponding PEC performance of the resulting thin films. In particular, we focused on evaluating the PEC stability to explore the potential of indium-organic complexes against photocorrosion. This approach offered an alternative strategy for the development of highly stable metal sulfides for a variety of light-induced applications, particularly for PEC water splitting.

## Experimental Section

### Chemicals

All chemicals were used as received without further purification, including iron­(III) chloride anhydrous (FeCl_3_, ≥97%, laboratory reagent grade, Fisher Scientific), l-cysteine hydrochloride anhydrous (C_3_H_8_ClNO_2_S, 97%, Thermo Scientific), indium­(III) chloride anhydrous (InCl_3_, 99.99%, metal basis, Thermo Scientific), sodium sulfide nonahydrate (Na_2_S·9H_2_O, ≥98.0%, Sigma-Aldrich), sodium sulfate anhydrous (Na_2_SO_4_, ≥99.0%, ReagentPlus, Sigma-Aldrich), and sodium sulfite anhydrous (Na_2_SO_3_, 98%, Thermo Scientific Chemicals).

### Synthesis of Indium Sulfide Thin Films and Powder

2 mmol of InCl_3_ and 6 mmol of cysteine hydrochloride were added to a 120 mL beaker, separately. FeCl_3_ (1 mmol) was weighed into a clean plastic weighing boat, and 60 mL of distilled water was poured over it to dissolve FeCl_3_ and transfer it completely to the same beaker. Upon addition, a transient blue coloration was observed at the center of the solution, which faded within seconds. The mixture was stirred using a magnetic stirrer for 10 min followed by 10 min of sonication. The resulting solution was transferred to a 100 mL Teflon-lined stainless-steel autoclave. A clean fluorine-doped tin oxide (FTO) substrate, with its top edge wrapped by a thermal tape, was vertically immersed in the solution with the conductive side facing the inner wall of the Teflon liner. The autoclave was sealed and placed in a Genlab oven at 160 °C for 6 h, using a ramp rate of 3 °C/min. After cooling to room temperature, the thin film was removed from the clear yellowish solution, rinsed several times with distilled water, and air-dried. The precipitation collected at the bottom of the autoclave was recovered by centrifugation, washed thoroughly with distilled water, and dried at 90 °C for 12 h.

The resulting samples were labeled based on their synthesis conditions as follows: 160–3 for samples prepared at 160 °C using a ramp rate of 3 °C/min for 6 h; 180–3 for samples prepared at 180 °C with the same ramp rate and duration time; and 160–10 for the sample prepared at 160 °C using a different oven (Memmert Oven) with a ramp rate of 10 °C/min for 6 h.

### Characterizations

The phase structure was analyzed using a Bruker D8 Advance X-ray diffraction (XRD) equipped with a Cu Kα radiation source (λ = 1.54 Å), operated at 40 kV and 40 mA. The corresponding data were collected in the 2θ range of 10 to 80°, with a step size of 0.05°. A Nexsa X-ray Photoelectron Spectrometer (XPS) from Thermo Fisher Scientific was used to analyze the surface chemical composition and chemical states, utilizing an Al Kα source with a wavelength (λ) of 1484.68 eV and a spot size of 400 μm by 400 μm. Survey spectra were recorded with a resolution of 200 eV, while high-resolution spectra were acquired at 40 eV. High-resolution transmission electron microscopy (HRTEM) images and selected area electron diffraction (SAED) patterns were obtained using a JEOL 2100 transmission electron microscope (JEOL Ltd., Japan) to examine the lattice fringes and determine the exposed crystallographic planes. The elemental analyses and corresponding atomic ratios were investigated by using a TESCAN VEGA3 scanning electron microscope (SEM) equipped with an Oxford X-MAXN energy-dispersive X-ray spectrometer (EDS) detector. A focused ion beam-scanning electron microscope (FIB-SEM), model Nova 600 Nanolab, manufactured by the FEI company (USA), was used to analyze both the top-view and cross-sectional morphology of the thin films. The optical properties of the thin films were investigated by using ultraviolet–visible diffuse reflectance spectroscopy (UV–vis DRS), recorded with a UV–vis-NIR spectrometer, manufactured by PerkinElmer (model Lambda 1050, USA).

### Photoelectrochemical (PEC) Characterization

Electrochemical measurements (Mott–Schottky analysis) were conducted using a CHI660E electrochemical workstation under the impedance-potential (IMPE) technique. A platinum (Pt) wire was used as the counter electrode, a saturated Ag/AgCl electrode was used as the reference, and the thin film sample was used as the working electrode. All three electrodes were immersed in a 0.5 M Na_2_SO_4_ electrolyte solution (pH 6.5). The measurement was carried out over a potential range from −1.0 to 0 V vs Ag/AgCl, with a potential increment of 5 mV and frequency of 1000 Hz. The electrochemical impedance spectroscopy (EIS) Nyquist plots were recorded at open-circuit potential (∼0.5 V vs Ag/AgCl) under continuous illumination. PEC analyses, including photocurrent density and photocurrent stability, were performed using an Autolab electrochemical workstation, model PGSTAT302N from Metrohm Autolab B.V. (Netherlands). Photocurrent density vs potential (*J*–*V*) plots were obtained using linear sweep voltammetry (LSV) in potentiostatic mode, scanning from −1.0 to 0.4 V vs Ag/AgCl at a scan rate of 0.01 V/s. The measurements were performed in an electrolyte containing 0.025 M Na_2_SO_3_ and 0.025 M Na_2_S·9H_2_O (overall pH 12.3), under chopped simulated sunlight (AM 1.5, 1 sun) provided by a Newport solar simulator operated at 300 W. The photocurrent stability was investigated by recording photocurrent density vs time plots using a chronoamperometry (Δ*t* > 1 ms) procedure at −0.2 V vs Ag/AgCl, in the same electrolyte, under continuous simulated solar light irradiation for 2 h.

## Results and Discussion

As illustrated in Figure 1, the major diffraction peaks at 2θ values of 14.2, 23.3, 27.4, 28.7, 33.3, 43.6, and 47.7° in both samples can be indexed to the (111), (220), (311), (222), (400), (511), and (440) planes, respectively, confirming the formation of a cubic In_2.67_S_4_ phase (ICSD No. 202353). Additionally, the peaks observed at 26.6, 37.8, 51.5, and 65.6° in Figure 1a are attributed to the SnO_2_ phase from the FTO substrate. A broad hump in the 2θ range of 10–20°, present only in the thin film sample, indicates the presence of an amorphous component. The XPS survey scans in Figure S1 show no detectable Fe 2s or Fe 2p peaks, and the minor Fe signal (≤0.2 At%) observed in EDS spectra (Figure S2) is attributed to background noise, confirming the absence of any iron-related phase. In contrast, the powder samples in Figure 1b exhibit sharper and more intense peaks than the thin film samples, without any diffraction signals from the FTO glass, further confirming the purity of the formed In_2.67_S_4_ phase.

**Figure 1:**
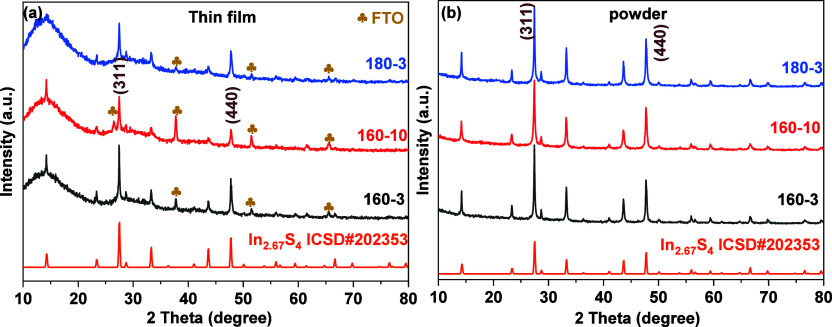
XRD patterns: (a) thin films and (b) corresponding powder.

The crystallite sizes of In_2.67_S_4_ thin films, calculated using the Scherrer equation,^38,39^ are listed in Table 1. Among the samples, thin film 160–3 exhibits the largest crystallite size at 105.9 nm—approximately twice that of thin film 180–3 (53.6 nm) and 2.5 times that of thin film 160–10 (43.0 nm). This indicates that thin films obtained at a lower temperature ramp rate 3 °C/min (160–3 and 180–3) tend to have larger crystallites compared with the thin film prepared at a higher ramp rate of 10 °C/min (160–10).

**Table 1:** Crystallite Sizes of In_2.67_S_4_ Thin Films Calculated Using the Scherrer Equation^38,39^

thin films	observed broadening/2θ	broadening of a standard sample/2θ	structural broadening/2θ	peak position/2θ	crystallite size/nm
**160–3**	0.2	0.118	0.082	47.75	105.9
**160–10**	0.32	0.118	0.202	47.74	43.0
**180–3**	0.28	0.118	0.162	47.76	53.6

According to the classic nucleation theory, the supersaturation ratio (*S*(*t*)) provides the fundamental driving force for crystallization:^40^

$$S(t)=\frac{C(t)}{C_{\mathrm{eq}}(T(t))}$$ 1

where *C*(*t*) is actual solute concentration released by precursor decomposition and *C*_eq_(*T*(*t*)) is the equilibrium solubility at temperature *T*(*t*).

The nucleation barrier (ΔG*), critical radius (r*), and nucleation rate (*B*_0_) are expressed as^40^

$$\Delta G^{*}=\frac{16\pi \sigma^{3}v^{2}}{3{\left(KT\ln S\right)}^{2}}$$ 2

$$r^{*}=\frac{2\sigma v}{KT\ln S}$$ 3

$$B_{0}=A\exp\left[\frac{-16\pi \sigma^{3}v^{2}}{3k^{3}T^{3}{\left(\ln S\right)}^{2}}\right]$$ 4

where σ is the surface tension, *v* is the molecular volume, *k* is the Boltzmann constant, *T* is the absolute temperature, *S* is the supersaturation ratio, and *A* is the pre-exponential factor.^40^ Both *T* and ln *S* influence the nucleation barrier; however, because ln *S* appears squared in the denominator of the exponent, variations in supersaturation have a far stronger effect on the nucleation rate than comparable changes in temperature. Therefore, supersaturation primarily governs the nucleation density and crystallite size.

At 160 °C with a faster ramp rate (10 °C/min), the precursor decomposition generates solute more rapidly than the equilibrium solubility (*C*(*t*) ≫ *C*_eq_(*T*(*t*))), leading to higher supersaturation. As ΔG* ∝ (1/ln *S*)^2^, the nucleation barrier decreases, yielding a higher nucleation rate; simultaneously, r* ∝ 1/ln *S*, resulting in smaller crystallites. In contrast, a slower ramp rate (3 °C/min) generates a lower supersaturation, which reduces the nucleation rate. Consequently, fewer nuclei form, and the solute released is distributed among a smaller number of growing crystals, allowing each to grow larger. Therefore, slower heating favors the formation of larger crystallites by promoting crystal growth over nucleation.

At the same ramp rate of 3 °C/min, the crystallites formed at 160 °C are larger than those at 180 °C. Although supersaturation provides the thermodynamic driving force for nucleation, the differences are primarily governed by reaction kinetics, which are strongly influenced by the temperature. At 160 °C, the equilibrium solubility (*C*_eq_(*t*)) is lower than that at 180 °C, initially producing higher supersaturation for the same amount of solute released. However, the lower temperature slows the reaction kinetics and limits the overall nucleation rate, resulting in fewer nuclei. With fewer nuclei competing for the available solute, each crystal has more material for growth, leading to larger crystallites. In contrast, at 180 °C, faster reaction kinetics and increased solubility promote the formation of more nuclei and greater competition for solute, resulting in a higher nucleation density and smaller crystallites. Additionally, the decomposition of sulfur sources, particularly l-cystine, occurs at 180 °C, further enhancing nucleation and contributing to the reduced crystallite size.^41,42^

Table 2 summarizes the nucleation behavior, crystallite grown, and proposed mechanistic explanations for each thin film.

**Table 2:** Nucleation Behavior and Growth Mechanisms of In_2.67_S_4_ Thin Films

thin films	supersaturation (*S*)	nucleation barrier (ΔG*)	critical radius (r*)	nucleation rate (*B*_0_)	crystallite size (nm)	proposed mechanistic explanation
160–3	low	high	large	low	large	slow heating → fewer nuclei → more solute per nucleus → larger crystallites
160–10	high	low	small	very high	small	fast heating → many nuclei → less solute per nucleus → smaller crystallites
180–3	moderate	moderate	moderate	higher than 160–3	moderate	higher temperature → faster reaction kinetics + l-cystine decomposition → more nuclei → moderate crystallite growth

The relative intensity ratios of the peaks located at 2θ values of 27.4 and 47.7° were calculated to estimate the corresponding facet ratios of (311)/(440). As summarized in Table 3, the facet ratio is 1.38 for thin film 160–3, 2.13 for 160–10, and 1.29 for 180–3, indicating that all thin films predominantly expose the (311) facet. These results are further supported by TEM analysis. The HRTEM images in Figure S3 reveal clear lattice fringes with interplanar spacings corresponding to the (311) plane in the samples, confirming the preferential exposure observed from XRD analysis. In addition, the selected area electron diffraction (SAED) patterns show well-defined diffraction rings indexed to the (311), (220), and (440) planes. For the thin film 160–10, the SAED pattern also exhibits a relatively strong contribution from the (111) plane, indicating a greater portion of this orientation. Notably, the thin film (160–10) synthesized at the higher ramp rate of 10 °C/min displays a relatively stronger (311) orientation compared with those prepared at the lower ramp rate of 3 °C/min, as reflected by the increased (311)/(440) intensity ratio. This variation is likely due to the reduced time for atomic diffusion and rearrangement under rapid heating conditions, which favors the development of kinetically preferred growth directions, with the (311) facet being dominant, rather than the thermodynamically stable (440) orientation. While the XRD result indicates that the (311) plane remains predominant, the increased peak intensity at 14.2° along with the HRTEM and SAED results have revealed that the (111) plane is also significantly developed. Therefore, the higher ramp rate not only enhances the relative nucleation of the (311) facet but also promotes the growth of the (111) planes, which contributes to the overall crystallographic texture of the thin film.

**Table 3:** Intensity Ratios of the (311) to (440) Diffraction Peaks in In_2.67_S_4_ Thin Films

thin film	(311) peak height/counts	(440) peak height/counts	(311)/(440)
**160–3**	723	524	1.38
**160–10**	456	214	2.13
**180–3**	474	367	1.29

Without involving FeCl_3_, thin films prepared at a ramp rate of 3 °C/min exhibited a (311)/(440) facet intensity ratio of 1.64, while those synthesized at 10 °C/min showed a ratio of 1.98.^28^ Introducing Fe^3+^ at 3 °C/min reduced the ratio from 1.64 to 1.38, suggesting that, although crystal growth at this slow ramp rate is primarily governed by thermodynamic factors, the oxidation of l-cysteine to l-cystine, which reduces thiol availability, further promotes growth along the thermodynamically favored (440) facet. At 10 °C/min, Fe^3+^ increased the (311)/(440) ratio from 1.98 to 2.13. At this faster ramp rate, the kinetic advantage of the (311) facet outweighs the thermodynamic stability of the (440) facet, so the reduced thiol availability does not inhibit (311) growth.

The high-resolution S 2p spectra shown in Figure 2a–c were fitted into three spin–orbit doublets, each exhibiting a binding energy separation of 1.18 eV between the S 2p1/2 and S 2p3/2 components.^43,44^ In Figure 2a, corresponding to the thin film 160–3, the first doublet (red) with an S 2p3/2 located at 161.21 eV is attributed to In–S bonding within the In_2_S_3_ lattice. The second doublet (green), at 163.41 eV (S 2p3/2) and 164.59 eV (S 2p1/2), is assigned to organic sulfur species (R–SH or C–S–S–C), likely originating from the In-cysteine or In-cystine complexes.^45^ The third doublet, centered at 168.07 eV (S 2p3/2) and 169.25 eV (S 2p1/2), is associated with the oxidized sulfur group, possibly the sulfonic acid group (R–SO_3_H), resulting from partial oxidation of thiol groups. The ratio of In–S:C–S–S–C/C-SH:R-SO_3_H is 36:18:1, indicating that lattice In–S contributes roughly twice as much as the organic sulfur species. For thin film 160–10 (Figure 2b), prepared under a higher heating rate compared with 160–3, the S 2p3/2 peaks corresponding to In–S and C-SH/C–S–S–C species shifted slightly to lower binding energies, appearing at 161.13 and 163.37 eV, respectively. The ratio of In–S:C–S–S–C/C–SH:R–SO_3_H is 17:19:1, showing nearly equal contribution from lattice and organic sulfur, which indicates that faster heating favors the formation of organic sulfur species as the predominant sulfur species. Additionally, the binding energy of the doublet associated with oxidized thiol species (R–SO_3_H) slightly increased to 168.10 eV. For thin film 180–3, prepared at a higher temperature compared to 160–3, all three S 2p spin–orbit doublets exhibited shifts toward higher binding energies. The S 2p3/2 component attributed to In–S located at 161.51 eV, while those corresponding to R–SH/C–S–S–C and R–SO_3_H were observed at 163.67 and 168.54 eV, respectively. The corresponding doublet ratio of In–S:C–S–S–C/C–SH:R-SO_3_H is 41:11:1, indicating that In–S is nearly four times higher than C–S–S-C/R-SH, reflecting a strong enrichment of lattice In–S with a reduction in organic sulfur contributions. Thus, heating rate and temperature significantly influence the balance between lattice In–S and organic sulfur species in the thin films.

**Figure 2:**
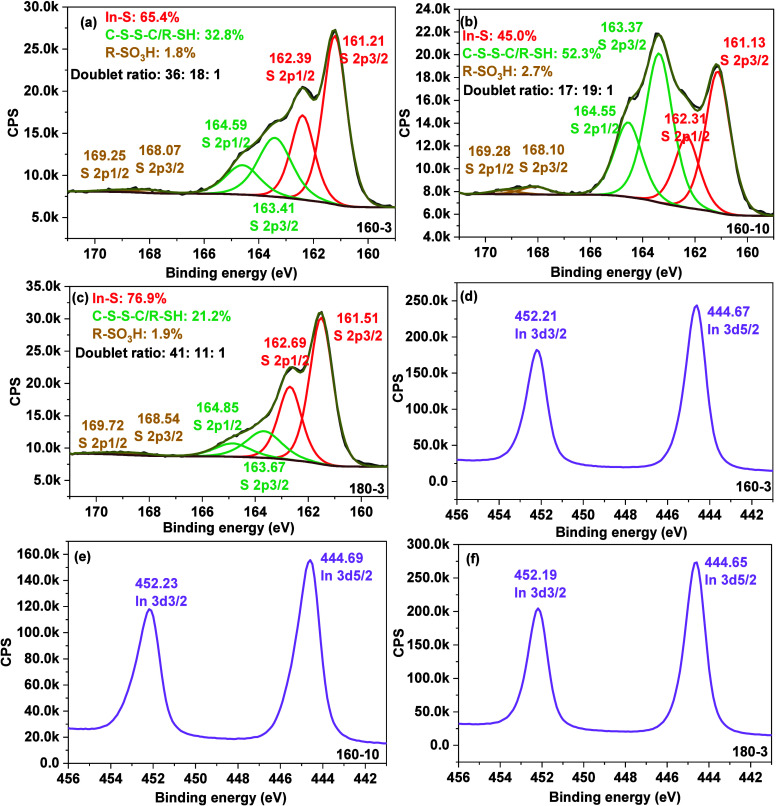
XPS high-resolution spectra of S 2p for (a) 160–3, (b) 160–10, and (c) 180–3 and In 3d for (d) 160–3, (e) 160–10, and (f) 180–3.

The existence of the oxidized thiol species indicates that the hydrothermal process proceeds within an oxidation environment. Initially, Fe^3+^ is reduced to Fe^2+^ through the oxidation of l-cysteine to l-cystine before the onset of the hydrothermal reaction.^45^ However, the oxidative environment can reoxidize Fe^2+^ to Fe^3+^, which then continues to oxidize the free thiol groups (R–SH) in l-cysteine to l-cystine until these free thiols are fully consumed. The S 2p results show that In–S species dominate the sulfur chemistry at the slower heating rate (3 °C/min), whereas organic sulfur species dominate at the faster heating rate (10 °C/min). This variation may also reflect the differences in the organic sulfur species formed, as faster heating likely limits the time available for the redox cycle from fully proceeding, resulting in a higher proportion of organic sulfur species associated with the In-cysteine rather than the more oxidized and stable In-cystine.

The high-resolution In 3d XPS spectra in Figure 2d–f were fitted with two distinct peaks corresponding to In 3d5/2 and In 3d3/2, resulting from the spin–orbit splitting of the In 3d orbital. The energy separation between these peaks was fixed at 7.54 eV, with a peak area ratio of 3:2 (In 3d3/2: In 3d5/2), which is consistent with the expected spin–orbit characteristics of indium.^43,44^ The binding energy of In 3d5/2 was nearly identical across the three films, varying only within a narrow range of 0.02–0.04 eV. Specifically, the In 3d5/2 peaks were located at 444.67 eV for the thin film 160–3, 444.69 eV for 160–10, and 444.65 eV for 180–3. This minimal variation indicates that the chemical state of indium remains unchanged among the samples and can be attributed to the In^3+^ presence in both the indium sulfide and indium-organic complexes. The high-resolution C 1s spectra (Figure S4­(a–c)) of all the thin films were fitted into four distinct peaks. The peaks located at 284.75 eV for thin films 160–3 and 160–10 and at 284.79 eV for 180–3 are attributed to C–C from both surface In-organic complexes and adventitious carbon.^46–48^ The peaks at 286.07 eV (160–3), 286.05 eV (160–10), and 286.12 eV (180–3) can be assigned to C–O bonds in both adventitious carbon and In-organic complexes, as well as C–S bonds originating from In-organic complexes.^46–48^ The peaks at 287.55 eV for 160–3 and 160–10 and 287.59 eV for 180–3 correspond to C–N bonds within the In-organic complexes.^46–48^ Finally, the peaks at 288.58 eV (160–3), 288.64 eV (160–10), and 288.69 eV (180–3), are attributed to C=O groups from both carboxylate (−COO^–^) and carboxylic acid (−COOH) in the In-organic complexes capping the surface of indium sulfide.^46–48^ The high-resolution O 1s spectra of all thin films, shown in Figure S4­(d–f), were fitted into four different peaks. From low to high binding energy, the first peaks located at 529.51 eV (160–3), 529.30 eV (160–10), and 529.52 eV (180–3) are attributed to lattice oxygen in metal oxides, most likely originating from M-O/Na auger in the substrate. The second peaks at 531.49 eV for 160–3, 531.55 eV for 160–10, and 531.41 eV for 180–3 are assigned to oxygen species in the carboxylate group (−COO^–^) presented in the surface-bound In-organic complexes. The third peaks, observed at 532.08 eV (160–3), 532.17 eV (160–10), and 531.99 eV (180–3), correspond to oxygen in the carboxylic acid groups (COOH) associated with the surface-capped In-organic complexes. The fourth peaks appearing at 533.40 eV (160–3), 533.39 eV (160–10), and 533.49 eV (180–3) are attributed to hydroxyl groups (O–H) from COOH species associated with the surface-bound In-organic complexes. The high-resolution N 1s XPS spectra of the prepared thin films were fitted with two components. As illustrated in Figure S4­(g–i), the first component located at 399.98 eV for 160–3, 400.02 eV for 160–10, and 399.92 eV for 180–3, is attributed to the amino groups (−NH_2_) in the surface-bound In-organic complexes.^49,50^ The second component, observed at 401.51 eV for both 160–3 and 180–3, and at 401.59 eV for 160–10, corresponds to the protonated amine groups (−N^+^) presented in the In-organic complexes.^49–51^

At low magnification, all thin films exhibit a relatively uniform porous morphology and good coverage, indicating successful film deposition. The morphology of thin film 160–3, shown in Figure 3(a–c), appears sponge-like, characterized by highly porous, loose, and interconnected network structures, with pores that vary in size and shape. In contrast, thin film 160–10 exhibits a textured morphology rather than a sponge-like structure, Figure 3(d–f). These textures are denser and finer with smaller and more irregular pores. The morphology difference between these two thin films is strongly influenced by the balance between nucleation density and subsequent crystal growth. 160–3 with fewer nuclei, crystal growth dominates, producing larger crystallites that interconnect into a loose, sponge-like porous network with pores of variable size and shape. In contrast, 160–10 with higher nucleation density gives rise to numerous small crystallites that grow competitively. The resulting microstructure is denser and more compact with finer and more irregular pores, consistent with the observed textured morphology.

**Figure 3:**
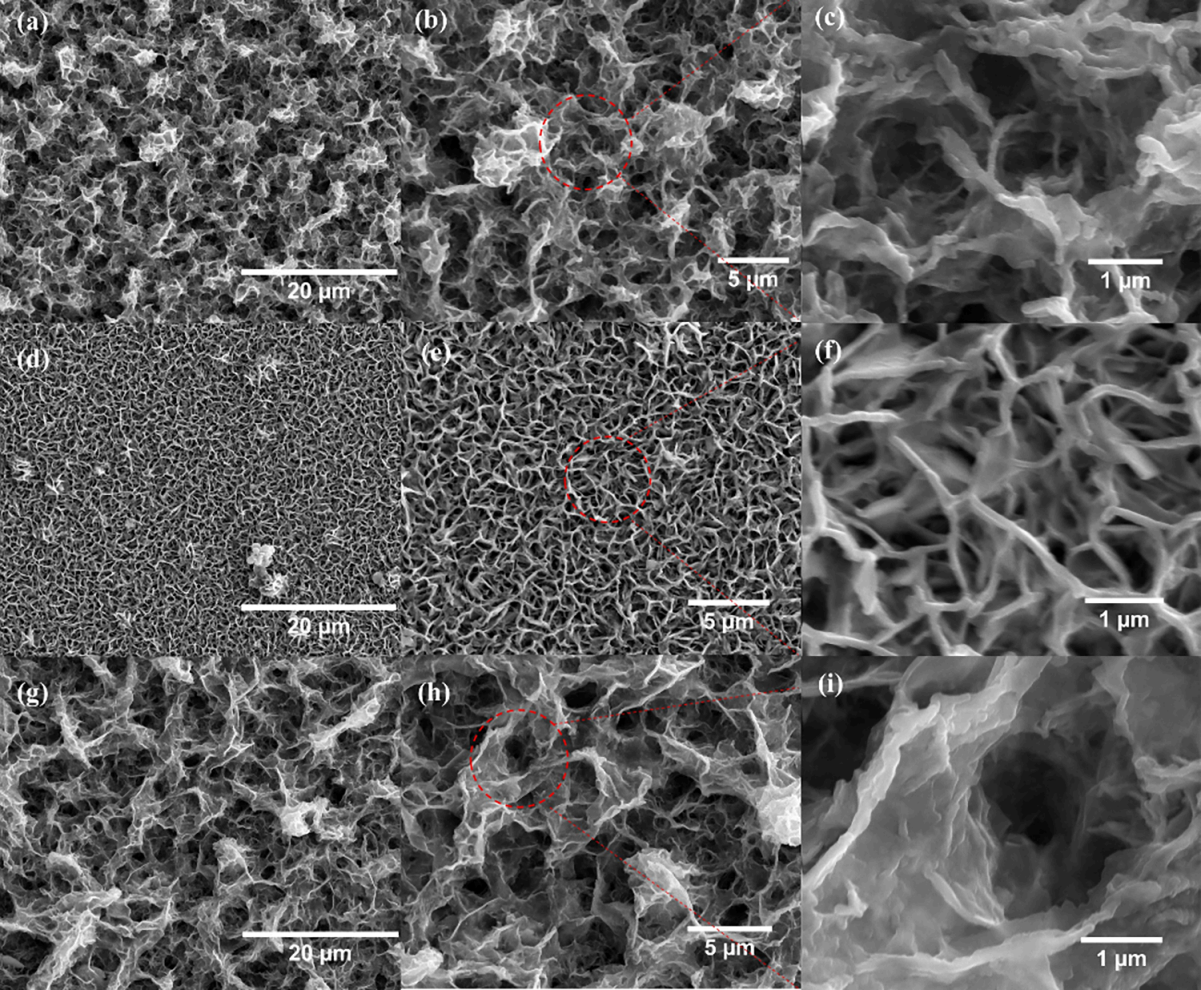
Low- and high-resolution SEM images of the obtained thin films: (a–c) 160–3, (d–f) 160–10, and (g–i) 180–3.

With or without FeCl_3_ addition in the hydrothermal precursor, the thin films exhibit obvious differences, as shown in Figure S5. Both thin films subjected to slower heating (Figure S5a,c) exhibited sponge-like structures; however, the introduction of FeCl_3_ resulted in a more uniform morphology with larger pore sizes. In contrast, films formed under faster heating conditions showed significant morphological differences: the thin film without FeCl_3_ displayed a petal plate-like assembly structure, whereas the thin film with FeCl_3_ exhibited a textured structure. These morphological differences between the indium sulfide thin films synthesized with and without FeCl_3_ arise from the redox cycling of Fe^3+^/Fe^2+^ in the precursor solution, which alters the balance between l-cysteine hydrochloride and l-cystine ligands. This shift in ligand speciation influences the complexation behavior of In^3+^ and the nucleation kinetics of the thin films. Specifically, under the same hydrothermal ramp rate, the introduction of FeCl_3_ leads to the coexistence of l-cysteine hydrochloride and l-cystine, creating a more complex and heterogeneous ligand environment. The formation of more stable In-cystine complexes and a reduced proportion of In-cysteine complexes slows the nucleation process and promotes more uniform film growth. In contrast, in the absence of FeCl_3_, the simpler ligand environment dominated by l-cysteine facilitates faster nucleation and less controlled growth, resulting in distinct morphological features. The FeCl_3_-containing system thus supports more gradual nucleation and growth than the FeCl_3_-free system, with these differences becoming more significant under rapid heating conditions.

Under slow heating but higher temperature (180 °C, 3 °C/min) conditions, the 180–3 thin film exhibits a similar sponge-like structure to that of the 160–3 thin film (160 °C, 3 °C/min), but with noticeably enlarged pores, thinner pore walls, and smaller crystallite sizes, as verified by the XRD results. These differences are likely due to the higher temperature accelerating precursor decomposition and reaction kinetics, which increases the nucleation rate. At 180 °C, the faster nucleation generates a greater number of smaller crystallites that grow simultaneously, consuming the available solute more evenly and producing thinner pore walls. Meanwhile, the lower nucleation density at 160 °C allows fewer larger crystallites to dominate, forming thicker walls and smaller pores. As a result, although both films retain a sponge-like morphology due to the slow heating rate, the thin film 180–3 exhibits larger pores and thinner walls, consistent with the smaller crystallite size observed in the XRD analysis.

The cross-sectional views in Figure 4 show that all thin films possess porous structures, consistent with the top-view image. Thin films 160–3 and 180–3 exhibit a sponge-like structure, whereas thin film 160–10 displays a textured structure composed of vertically grown nanosheets. The thin film prepared via fast heating (160–10) has the smallest thickness, measuring 2.0 μm, while the thin film synthesized under slow heating at the same hydrothermal temperature (160–3) is thicker, with a thickness of 9.6 μm. This difference can be attributed to the effect of nucleation and growth kinetics. Slow heating (3 °C/min) allows a lower nucleation rate and more time for individual crystallites to grow, leading to thicker films. In contrast, fast heating (10 °C/min) induces rapid nucleation, generating a high density of small crystallites that compete for the available solute, which limits vertical growth and results in a thinner film. Further increasing the hydrothermal temperature under slow heating (180–3) produces an even thicker film of 13.8 μm, likely due to the accelerated reaction kinetics and enhanced precursor decomposition at 180 °C, which supply a larger number of reactive species. Under slow heating, the lower nucleation density allows these species to be consumed by fewer nuclei, promoting extensive vertical growth and resulting in a thicker film. Additionally, the faster kinetic at higher temperatures facilitate more rapid crystal growth, further contributing to the increased film thickness compared with thin film 160–3.

**Figure 4:**
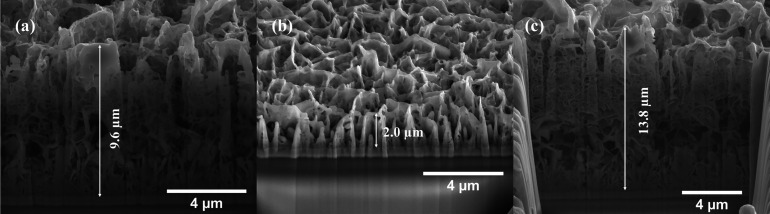
Cross-sectional SEM images of the prepared thin films: (a) 160–3, (b) 160–10, and (c) 180–3.

The UV–vis DRS results shown in Figure 5a indicate that the reflectance intensity of thin film 180–3 is the highest among the three samples followed by those of 160–3 and then 160–10. This suggests that the sponge-like morphology of 160–3 and 180–3 enhances the light reflection compared with the more compact texture network observed in 160–10. Moreover, in comparison with 180–3, the thin film 160–10 exhibits a significant red shift, while 160–3 shows a slight red shift. These shifts are likely attributed to the differences in chemical composition resulting from variations in heating rate and temperature. As discussed in the XPS results, thin film 160–10 contains indium-complexes as the dominant surface components, whereas both 160–3 and 180–3 primarily consist of indium sulfide, with 180–3 having a higher indium sulfide ratio. The presence of indium-complexes on the surface of all the thin films may modulate the band structure, thereby influencing their band gap values. As shown in Figure 5b–d, the band gaps estimated using the Kubelka–Munk function^52^ are 2.54 eV for 160–3, 2.38 eV for 160–10, and 2.49 eV for 180–3. These results are consistent with the surface composition analysis, where a higher concentration of indium-complexes, particularly in thin film 160–10, is associated with a narrower band gap, suggesting that these surface species play a significant role in the band gap modulation.

**Figure 5:**
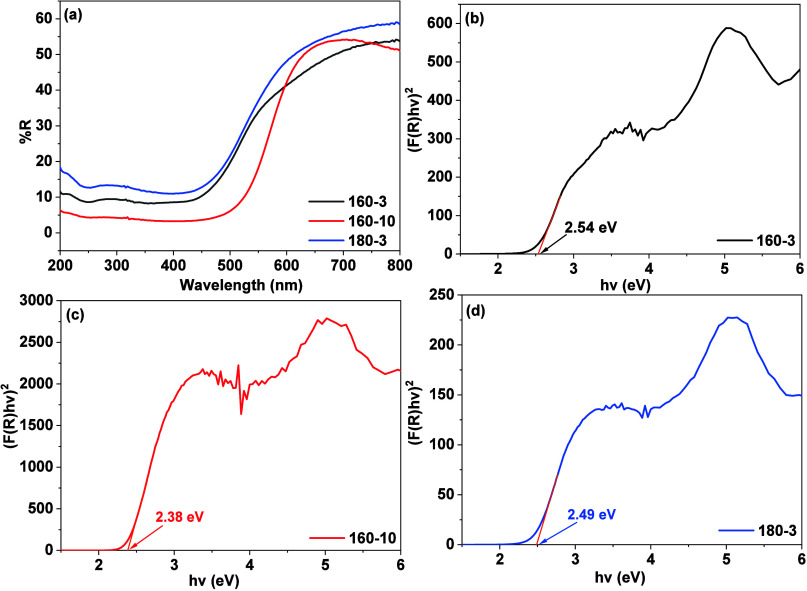
UV–vis DRS results of all the thin films (a), and their corresponding (F­(R)­h*v*)^2^ vs *hv* plots derived from the Kubelka–Munk function: (b) 160–3, (c) 160–10, and (d) 180–3.

The Mott–Schottky plots of all thin films, shown in Figure 6, were analyzed to investigate their semiconductor properties through flat band potential determination and to explore the interface charge behavior. All thin films show two distinct positive linear regions, with a noticeable hump in between. The positive slopes of these linear regions confirm that the thin films exhibit n-type semiconductor behavior. The hump between the two regions is likely attributed to the reduction of surface-capped In-cystine complexes.^53–55^ Notably, for thin films 160–3 and 160–8, the first linear region appears between −1.0 and −0.85 V vs Ag/AgCl, while the second linear region starts from −0.5 to 0 V vs Ag/AgCl. In contrast, thin film 160–10 displays the first linear region from −1.0 to −0.75 V vs Ag/AgCl followed by a similar second region from −0.5 to 0 V vs Ag/AgCl. This shift may be due to incomplete precursor conversion caused by the rapid heating, resulting in In-organic complexes remaining as the dominant component. Compared with thin films 160–3 and 180–3, the shift observed in thin film 160–10 may indicate the presence of both In-cystine and In-cysteine complexes, where the observed hump could stem from the partial reduction of cystine to cysteine during the measurement. The flat band potential was extracted from the first linear region, as it occurs before the redox processes begin to influence the interfacial capacitance.^53–55^ The obtained flat band potentials (vs RHE) were −0.36 V for 160–3, −0.31 V for 160–10, and −0.40 V for 180–3. The flat band potential of thin film 160–10 shifted to a more positive value, likely due to the dominance of indium-organic complexes (In-cystine and In-cysteine) on the surface. Moreover, the Mott–Schottky plot for thin film 160–3 exhibited the highest 1/C^2^ values on the *y*-axis followed by thin film 160–10 and then 180–3. This trend indicates that thin film 160–3 possesses the lowest capacitance and, consequently, the widest depletion region.^56^ A wider depletion region would enhance the electric field strength across the junction, thereby improving the charge carrier separation efficiency.^56^ In comparison, thin film 180–3 exhibited the lowest 1/*C* ^2^ values, indicating the highest capacitance and narrowest depletion region.^56^ This narrower depletion region would reduce the electric field strength and limit the effective charge separation, which may negatively impact its photoelectrochemical performance.^56^ Thin film 160–10 had intermediate 1/*C* ^2^ values, reflecting a moderate capacitance and space charge width.^56^ The carrier densities of the thin films were calculated using eq 5,^57^ derived from the Mott–Schottky equation.^58^

$$N_{d}=\frac{2}{\varepsilon \varepsilon_{0}eS}$$ 5

where *N*_d_ is the carrier density (cm^–3^), *e* is the elementary charge (1.602 × 10^–19^ C), ε_0_ is the vacuum permittivity (8.854 × 10^–14^ F/cm), ε is relative dielectric constant of the In_2_S_3_ (taken as 8.5),^59^ and *S* is the slope of the linear region in the Mott–Schottky plot. Based on the slope values extracted from Figure 6, the estimated carrier densities (*N*_d_) are 1.43 × 10^18^ cm^–3^ for thin film 160–3, 8.17 × 10^18^ cm^–3^ for 160–10, and 2.92 × 10^19^ cm^–3^ for 180–3. The variations in carrier density show a relationship with the width of the depletion region, where lower carrier densities correspond to larger space charge regions.

**Figure 6:**
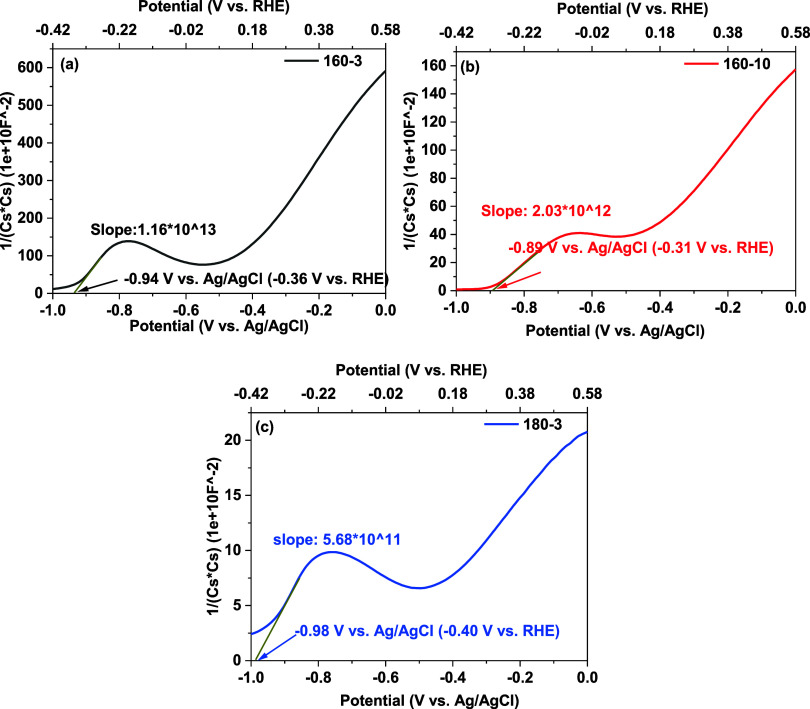
Mott–Schottky plots of the thin films: (a) 160–3, (b) 160–10, and (c) 180–3.

In our work, a potential difference of 0.1 V between the conduction band potential and the flat band potential was adopted, consistent with the reports indicating that the conduction band potential of n-type semiconductors is typically more negative than the flat band potential by approximately 0–0.2 V.^59^ As the potential difference between the conduction band and valence band corresponds to the value of the band gap energy, the valence band potentials of all thin films were determined accordingly. Figure 7a presents the band structures of all thin films under pH 6.5 vs the reversible hydrogen electrode (RHE). Using the conversion tool developed by the McAuley Group,^60^ we can derive the band structure at pH 0 vs RHE, Figure 7b. It is worth noting that under pH 0 the potential difference between V vs RHE and V vs NHE is negligible (within 0.01 V), and thus they are considered equivalent. As shown in Figure 7, all the thin films possess the capabilities for overall water splitting, as their conduction band potentials are more negative than the water reduction potential (H^+^/H_2_), and their valence band potentials are more positive than the water oxidation potential (H_2_O/O_2_). The film 160–3 has the most positive valence band potential, while the thin film 180–3 has the most negative conduction band potential. Thin film 160–10 has both conduction and valence band potentials positioned between these two thin films. These differences may further contribute to the variations in the PEC performance.

**Figure 7:**
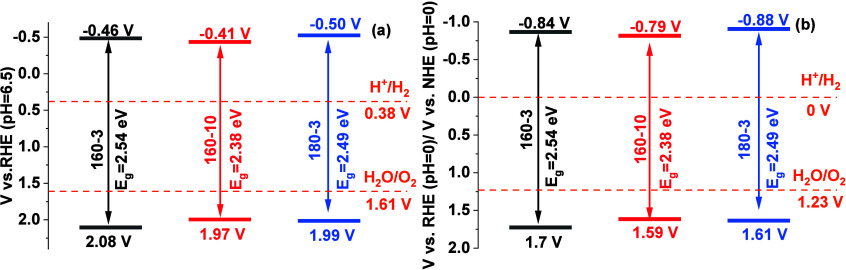
Schematic of the band structures and electrochemical potential of the prepared thin films at different pH values: (a) pH 6.5 and (b) pH 0.

To investigate the variations in PEC performance, the *J*–*V* plots under chopped light illumination are shown in Figure 8a for thin films 160–3 and 180–3, and in Figure S6 for 160–10. Among the samples, thin film 160–3 shows the highest overall photocurrent followed by 180–3 and then 160–10. Within the potential range of −0.9 to −0.5 V vs Ag/AgCl, 160–10 exhibits a higher photocurrent density than both 160–3 and 180–3. From −1 to −0.8 V vs Ag/AgCl, thin film 160–10 displays an initial increase in the photocurrent density with upward steps features followed by a gradual decrease characterized by downward steps up to 0.1 V vs Ag/AgCl. Beyond 0.1 V vs Ag/AgCl, the steps become smoother and more stable. Given the scan rate of 0.01 V/s, the transition from upward to downward steps occurs within approximately 20 s, and from downward to smooth steps within around 90 s. These transitions suggest significant changes in the film’s resistance, likely due to photocorrosion-induced phase transformation. In contrast, the thin film 160–3 exhibits nearly consistent upward steps throughout the scan, suggesting a stronger PEC stability. For thin film 180–3, it displays a steady-state photocurrent with upward steps beyond −0.2 V vs Ag/AgCl, indicating only a minor photocorrosion occurred during the LSV scan.

**Figure 8:**
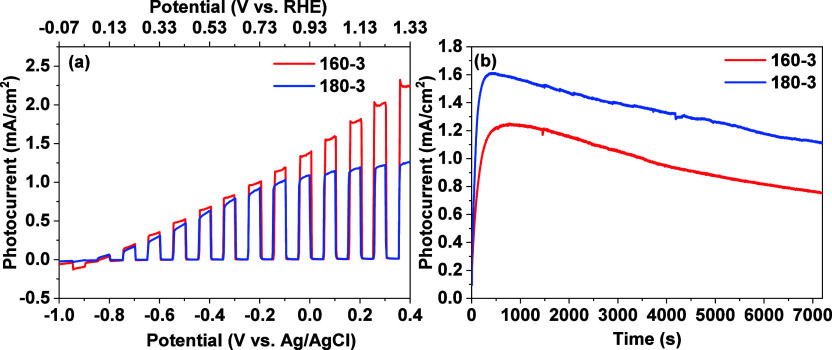
(a) *J*–*V* plots under chopped light illumination and (b) photocurrent stability under continuous light illumination at −0.2 V vs Ag/AgCl for thin films 160–3 and 180–3.

To further investigate the charge-transfer characteristics underlying the PEC behaviors, EIS Nyquist plots were analyzed. Figure S7 shows the EIS Nyquist plots of the thin films together with the corresponding fitted equivalent circuit. The equivalent circuit consists of a series resistance (*R*_1_), a bulk/substrate–film contribution (*C*_2_||*R*_2_), an interfacial charge-transfer element at the electrode–electrolyte interface (*Q*_3_||*R*_3_), and a low-frequency component (*C*_4_||*R*_4_) associated with slow interfacial processes.^57^ The fitted parameters are summarized in Table S1. The charge-transfer resistance (*R*_3_) follows the order 160–3 < 180–3 < 160–10, indicating progressively slower interfacial charge transfer, which is consistent with the observed photocurrent densities. Although thin film 180–3 exhibits a relatively high *Q*_3_ value, the presence of large *R*_4_ and *C*_4_ values indicates the involvement of deep trap states and slow recombination processes, which limits its PEC performance.

With the addition of FeCl_3_ in the hydrothermal precursor, thin film 160–10 exhibits a significantly lower photocurrent density compared with the film prepared without FeCl_3_ (LCHCl-IS-10, 160 °C, 10 °C/min), although both thin films display an upward-to-downward step transition.^28^ Similarly, when compared with LHCl-IS-3 (160 °C, 3 °C/min, without FeCl_3_), thin film 160–3 also shows reduced photocurrent density, but with nearly consistent upward steps.^28^ These differences in the PEC behavior likely originate from variations in the surface chemical composition. Thin film 160–10 likely contains both In-cystine and In-cysteine complexes, whereas 160–3 predominantly features In-cystine complexes.

To investigate the influence of In-cystine complexes on the PEC stability of indium sulfide, photocurrent density vs time plots were measured under continuous light illumination, as shown in Figure 8b. Both thin films 160–3 and 180–3 exhibit an initial increase in photocurrent density: thin film160–3 reaches its highest photocurrent density of 1.25 mA/cm^2^ at 783 s, while thin film 180–3 reaches 1.6 mA/cm^2^ at 367 s. Following this initial peak, both thin films experience a gradual decrease in the photocurrent density. After 2 h of illumination, thin film 160–3 retains 0.75 mA/cm^2^, while 180–3 maintains 1.1 mA/cm^2^. The initial boosting in photocurrent observed in these thin films can be attributed to the photooxidative cleavage of disulfide (C–S–S–C) bonds within In-cystine complexes.^61,62^ This process generates sulfur-centered radicals, which can be further converted into free sulfide ions (S^2–^). These sulfide ions can fill sulfur vacancies in the indium sulfide lattice that arise from light-induced degradation. Meanwhile, the exposed In^3+^ sites contribute to improving the charge transfer efficiency at the photoelectrode–electrolyte interface. This self-healing mechanism stabilizes the photoelectrode, explaining the observed initial increase in photocurrent followed by a gradual decrease as the supply of sulfide ions is exhausted.

Thin film 180–3 exhibits a rapid photocurrent rise and achieves a higher photocurrent density compared with thin film 160–3. This difference can be attributed to its lower content of In-cystine complexes bonded on the surface, which degrades more quickly than that in 160–3, allowing 180–3 to reach its steady state faster. Its smaller crystallites and highly porous structure provide more active sites for light absorption and charge separation, leading to the higher photocurrent density. During the fast LSV scan under chopped light, the larger crystallites of thin film 160–3 reduce grain boundary recombination, resulting in higher peak photocurrents due to the limited time for surface photocorrosion to develop. However, under steady illumination, the faster degradation of In-cystine on 180–3 and its more open structure promote better sulfur replenishment and charge transport, ultimately allowing 180–3 to outperform 160–3 after stabilization.

Without FeCl_3_, the thin films (LCHCl-IS-3 and LCHCl-IS-10) consist of In-cysteine-bonded indium sulfide, in which sulfur atoms exist as terminal thiol groups (C-SH);^28^ these thiol groups undergo photooxidation to form sulfur-centered radicals. However, these radicals remain covalently attached to the organic framework and cannot release free sulfide ions. Consequently, there is no sulfur replenishment mechanism available to repair the sulfur vacancies in the indium sulfide lattice. This absence of sulfur recovery leads to a rapid lattice destabilization, resulting in a fast decline in the photocurrent and eventual loss of photoactivity.

## Conclusions

In this study, we successfully synthesized (311)-oriented In-cystine bonded indium sulfide thin films with high PEC stability via a hydrothermal method using a mixed sulfur source of l-cysteine hydrochloride and in situ-generated l-cystine through Fe^3+^-mediated oxidation. We systematically investigated the effects of hydrothermal temperature and ramp rate on crystallite size, phase composition, morphology, optical properties, and PEC performance. Slow ramping (3 °C/min) favored sponge-like morphologies with indium sulfide as the dominant phase and surface bonding via In-cystine, whereas fast ramping (10 °C/min) produced denser, textured films dominated by In-cysteine and In-cysteine complexes. The thin films 160–3 and 180–3 exhibited a significantly enhanced PEC performance compared with 160–10, achieving photocurrent densities of 1.0 and 0.93 mA/cm^2^ at −0.2 V vs Ag/AgCl, respectively, vs only 0.35 mA/cm^2^ for 160–10. Increasing the hydrothermal temperature from 160 to 180 °C under slow ramping reduced crystallite sizes and enlarged surface pores, with 180–3 maintaining 1.1 mA/cm^2^ after 2 h of continuous illumination, compared with 0.75 mA/cm^2^ for 160–3. These results underscore the importance of controlled hydrothermal synthesis and the strategic incorporation of stable disulfide metal–organic complexes in improving the durability and performance of metal sulfide photoanodes. The In-cystine-bonded sulfide films offer a promising direction for the development of highly stable and efficient photoelectrode for solar-driven water splitting and other light-induced applications.

## Supplementary Material



## Notes

XPS survey and high-resolution scan; EDS spectra; HRTEM images with SAED patterns; SEM images; *J*–*V* plot of 160–10; EIS Nyquist plots; original cross-sectional SEM images (PDF)
