# Supplementary material for: Photoelectrochemical Stability Enhancement of (311)-Oriented Indium Sulfide Thin Films via In-Cystine Complex Formation under Hydrothermal Synthesis
Source: ACS Appl Energy Mater. 2026 Feb 10;9(4):2131–43. doi: 10.1021/acsaem.5c03482 (PMC12933517; doi:10.1021/acsaem.5c03482)
Supplement: Supplementary file 1 [file ae5c03482_si_001.pdf]

Supporting Information

Photoelectrochemical Stability Enhancement of (311)-Oriented Indium Sulfide Thin Films via In-Cystine Complex Formation under Hydrothermal Synthesis

Xiuru Yang[a], Hong Chang[a], Arthur Graf[b, c], Xiaohong Li[d], Yongde Xia[a], Asif Ali Tahir*[e], Yanqiu Zhu*[a]

[a] Department of Engineering, Faculty of Environment, Science and Economy, University of Exeter, Exeter, EX4 4QF, UK

[b] HarwellXPS, Research Complex at Harwell, Rutherford Appleton Lab, Didcot, OX11 0FA, UK

[c] School of Chemistry, Cardiff University, Main Building, Park Place, Cardiff, CF10 3AT, UK

[d] Renewable Energy Group, Department of Engineering, Faculty of Environment, Science and Economy, University of Exeter, Penryn TR10 9FE, UK

[e] Solar Energy Research Group, Environment and Sustainability Institute, Faculty of Environment, Science and Economy, University of Exeter, Penryn Campus, Penryn TR10 9FE, UK

*Corresponding authors

E-mail: y.zhu@exeter.ac.uk; A.Tahir@exeter.ac.uk

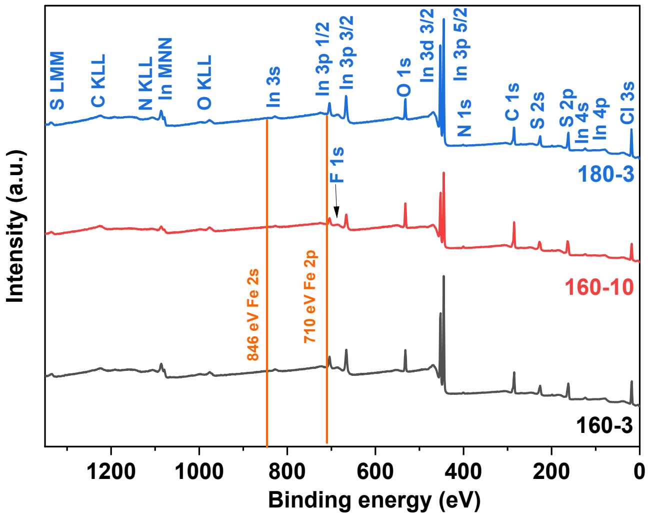

Figure S1. XPS survey scans of the produced thin films.

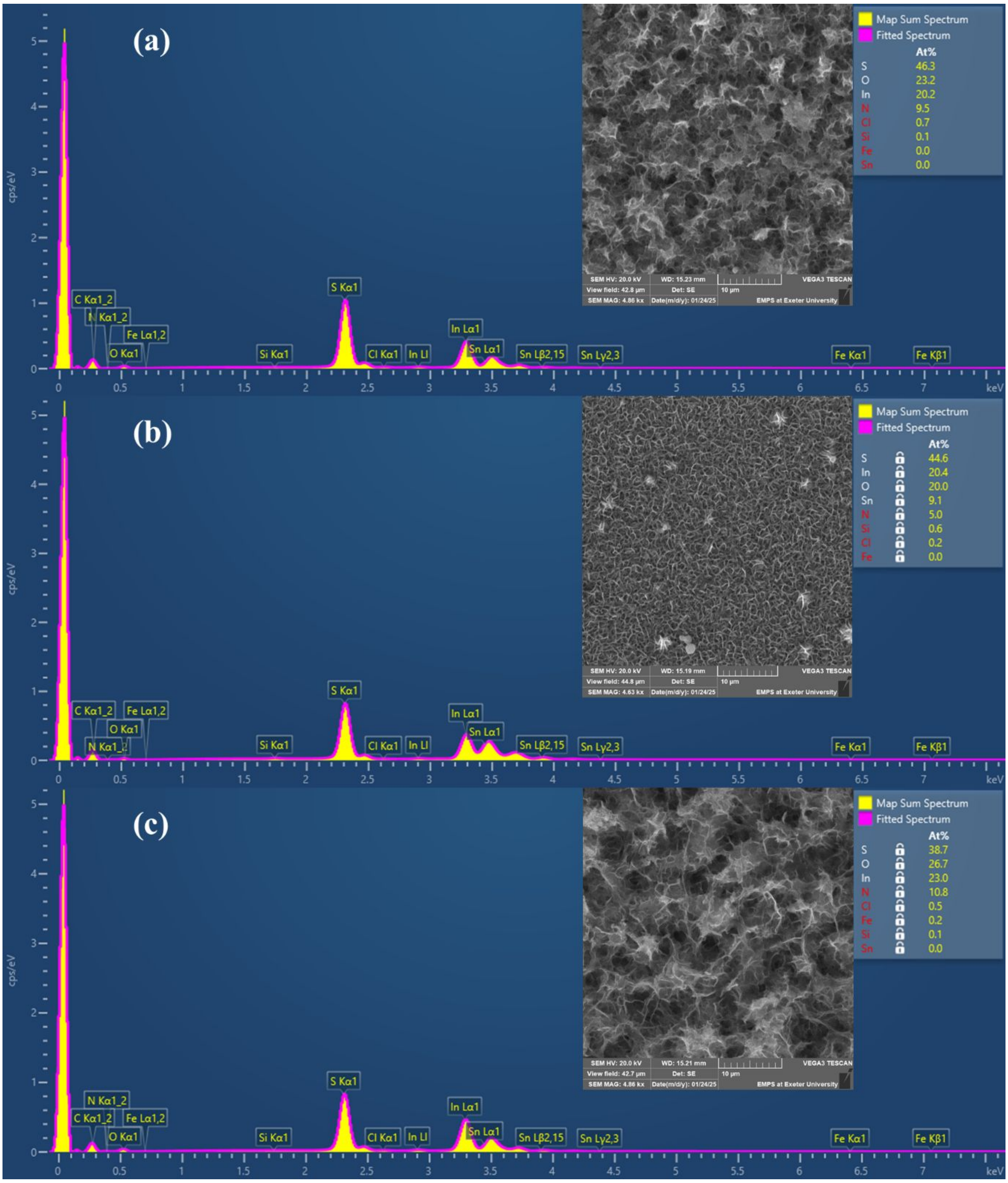

Figure S2. EDS spectra of the prepared thin films: (a) 160-3, (b) 160-10, and (c) 180-3.

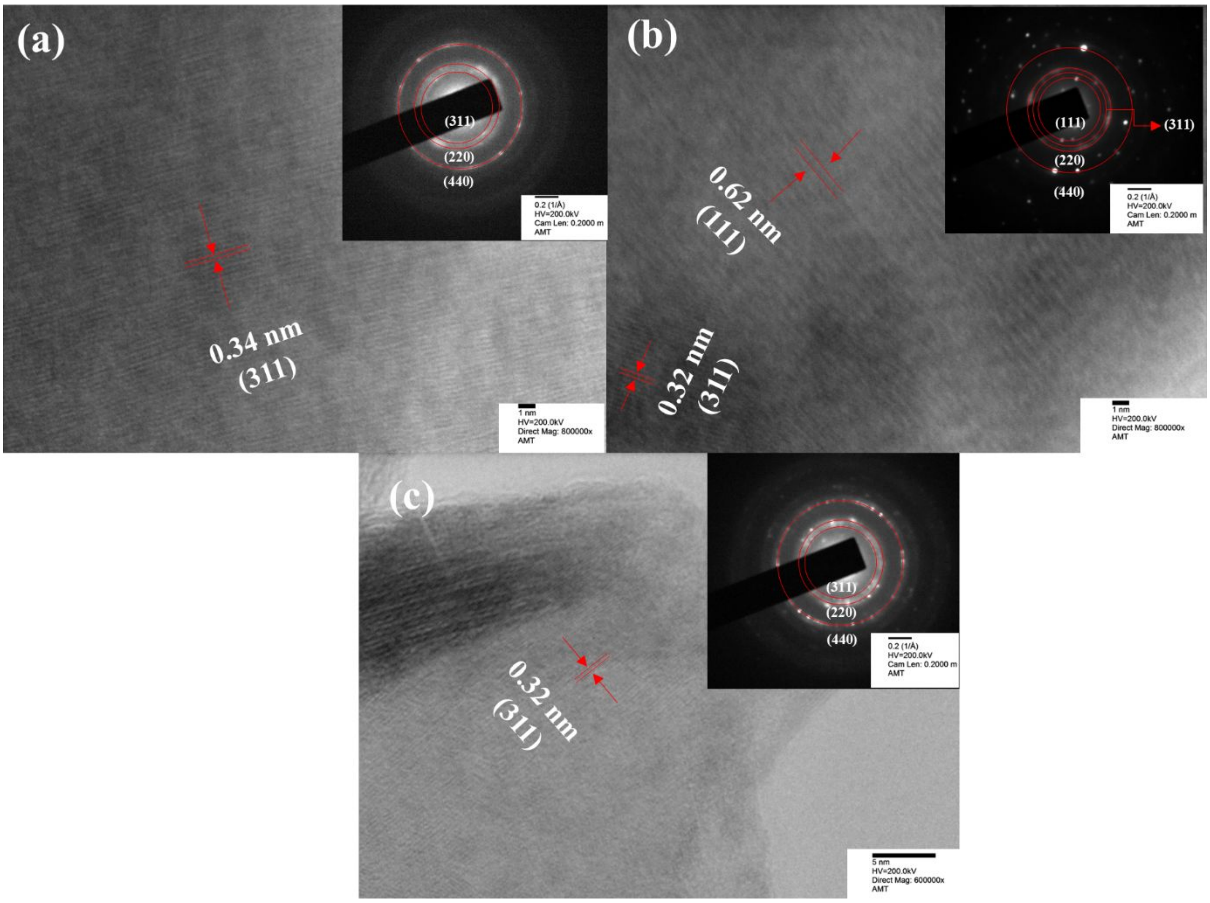

Figure S3. HRTEM images with insets showing the SAED patterns of the thin films: (a) 160-3, (b) 160-10, and (c) 180-3.

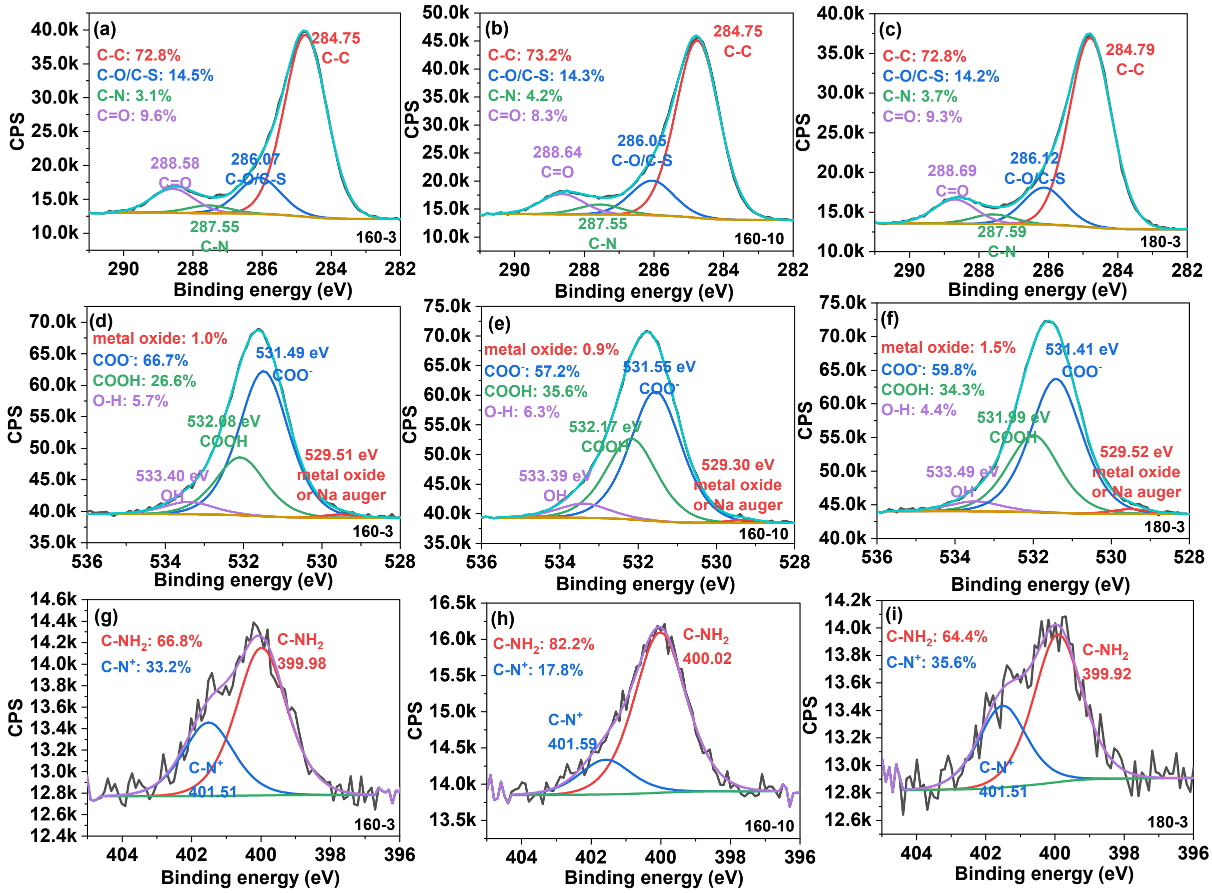

Figure S4. High-resolution XPS spectra of (a-c) C 1s, (d-f) O 1s, and (g-i) N 1s for thin films 160-3, 160-10, and 180-3, respectively: (a, d, g) 160-3; (b, e, h) 160-10; and (c, f, i) 180-3.

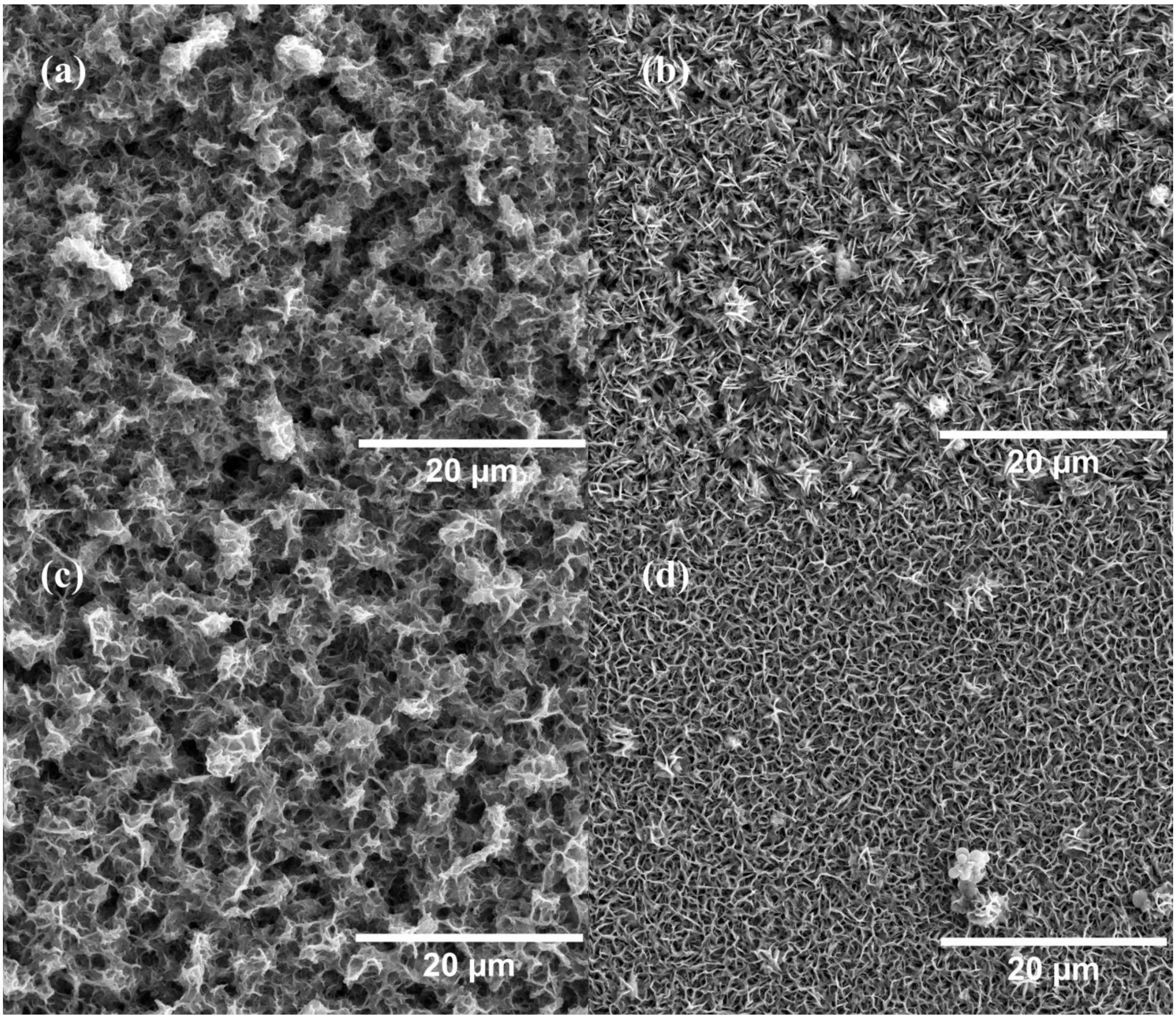

Figure S5. SEM images of prepared thin films: (a) LCHCl-IS-3, (b) LCHCl-IS-10, (c)160-3, and (d) 160-10.

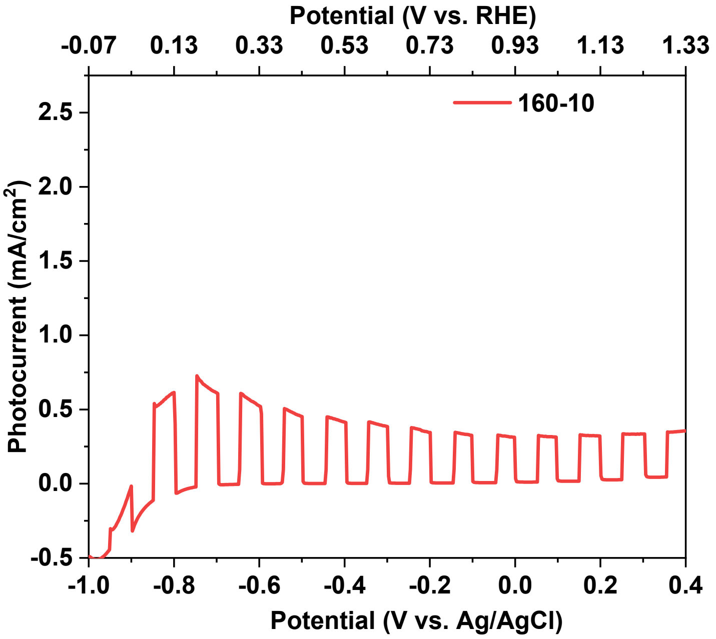

Figure S6. J-V plots for thin film 160-10 under chopped light illumination.

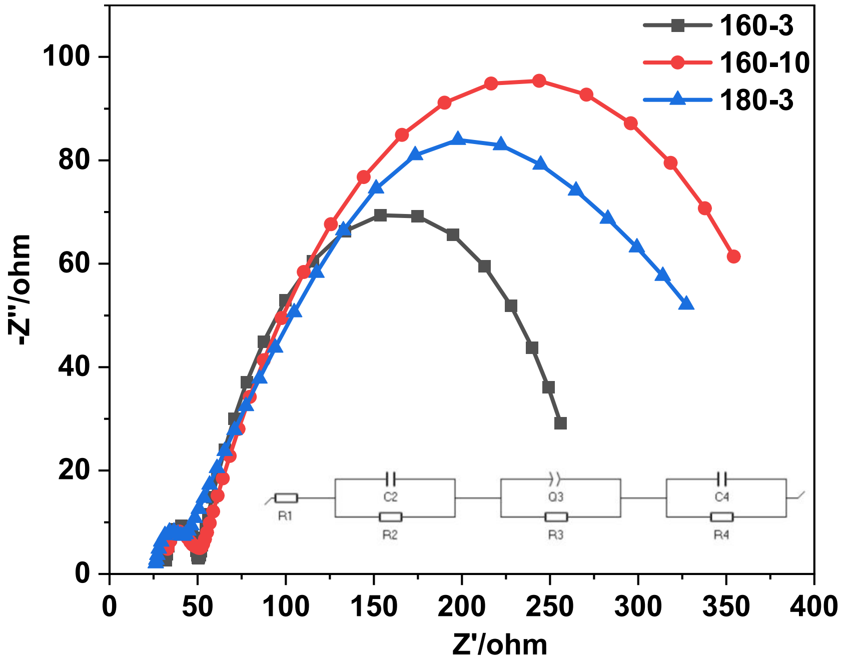

Figure S7. EIS Nyquist plots of thin films 160-3, 160-10, and 180-3 measured under illumination, with an inset showing the equivalent circuit.

Table S1. A summary of the Parameters of the Equivalent Circuit Used to Model the EIS Nyquist Plots.

Thin Film	R_1 (□)	R_2 (□)	C_2 (F)	R_3 (□)	Q_3 ($F*s^{(a-1)}$)	a	R_4 (□)	C_4
**160-3**	31.5	0.18	0.12	226.6	$1.0*10^{-3}$	0.7	18.05	$6.0*10^{-7}$
**160-10**	31.3	4.04	$1.0*10^{-5}$	369.7	$1.0*10^{-3}$	0.6	14.5	$3.2*10^{-7}$
**180-3**	25.0	10.2	$1.4*10^{-6}$	326.2	$1.5*10^{-3}$	0.4	58.23	$1.5*10^{-3}$

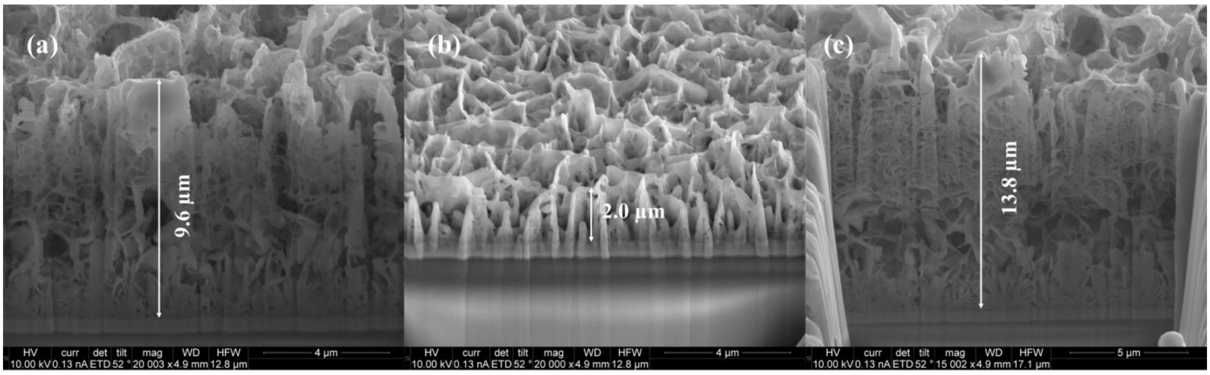

Figure S8. Original cross-sectional SEM images of the prepared thin films: (a) 160-3 (b) 160-10, and (c) 180-3.
